# Indocyanine green near-infrared imaging-guided lymph node dissection during oesophageal cancer surgery: A single-centre experience

**DOI:** 10.3389/fsurg.2022.982306

**Published:** 2023-01-09

**Authors:** Saihua Chen, Xiaofeng Tian, Guanjun Ju, Minxin Shi, Yibiao Chen, Qing Wang, Wencheng Dai, Tinghua Li, Jing Pan, Yihui Fan

**Affiliations:** ^1^ Department of Thoracic Surgery, Tumor Hospital Affiliated to Nantong University, Nantong Tumor Hospital, Nantong, China; ^2^ Department of Endoscopy Center, Tumor Hospital Affiliated to Nantong University, Nantong Tumor Hospital, Nantong, China; ^3^ Department of Head and Neck Surgery, Tumor Hospital Affiliated to Nantong University, Nantong Tumor Hospital, Nantong, China

**Keywords:** indocyanine green, near infrared, esophageal cancer, lymphadenectomy, metastatic nodes

## Abstract

**Objective:**

This study aimed to investigate the feasibility of using indocyanine green (ICG) near-infrared (NIR) imaging during lymphadenectomy for oesophageal cancer.

**Methods:**

Eighty-seven patients with primary oesophageal cancer were enrolled in this study. All the enrolled patients received an endoscopic injection of ICG between 40 min and 23 h before surgery. Nodal dissection during surgery was performed under fluorescence imaging visualisation, with the NIR signal shown in purple. ICG^+^ or ICG^−^ nodes were recorded station by station and were microscopically evaluated.

**Results:**

Endoscopic peritumoral ICG injection was successfully performed in all patients. Major post-surgery complications included wound infection, pleural effusion, dysphonia, pneumonia and anastomotic fistula. No patients experienced ICG-related adverse events. A total of 2,584 lymph nodes were removed, and the mean number of lymph nodes for each patient was 29.70 ± 9.24. Most of the removed nodes (97.83%) were ICG^+^, and 3.32% of the ICG^+^ nodes were metastatic. No metastatic nodes were ICG^−^ or belonged to an ICG^−^ lymph node station. The time from ICG injection to surgery did not affect the number of harvested lymph nodes.

**Conclusions:**

The use of ICG-NIR imaging during oesophageal cancer surgery can enhance the visualisation of lymph nodes during surgery. It is a feasible, safe and helpful technique for lymphadenectomy.

## Introduction

Oesophageal cancer is one of the most aggressive gastrointestinal malignancies, with a 5-year overall global survival rate of 15%–25% (1, 2). It is the eighth most common cancer type and the sixth leading cause of death from cancer in the world (3). The incidence of oesophageal cancer is increasing year on year (4).

Surgical or endoscopic resection remains the mainstay of treatment for oesophageal cancer (5). It has been reported that lymphadenectomy can significantly improve the long-term survival and accuracy of tumour staging in patients with oesophageal cancer (6). Extensive lymphadenectomy was associated with improved prognosis (6). Currently, lymphadenectomy is often performed using a surgeon's experience without the aid of visual instruments. However, due to the complex lymphatic drainage around the oesophagus, it is a challenge for surgeons to distinguish which lymph nodes are at risk of metastatic disease. To remove as many lymph nodes as possible and decrease the risk of recurrence, the removal of ≥20 lymph nodes for T_2_ disease and ≥30 for T_3_/T_4_ disease has been suggested (7, 8). However, even an extensive 3-field lymphadenectomy may miss some key nodes containing occult metastases, resulting in a 5% risk of disease recurrence (6). Compared with 3-field lymphadenectomy, the commonly used 2-field lymphadenectomy technique has an even higher risk of recurrence (9). Thus, finding a method to dissect sufficient lymph nodes efficiently and accurately is important for the development of lymphadenectomy.

Near-infrared (NIR) fluorescence-guided imaging has been proven to enable surgical visualisation beyond that possible with the naked eye (10). Indocyanine green (ICG) is a Food and Drug Administration-approved NIR dye with a fluorescence emission wavelength ranging from 800 to 1,000 nm (11). The peak absorption and emission wavelengths of ICG are 785 and 810 nm, respectively (11). Indocyanine green-NIR is a surgical navigation technique that has achieved satisfactory results in the localisation of lymph nodes in patients with breast cancer (12), gastric cancer (13), non-small cell lung cancer (14) and other cancers (15). Some studies have been published on the application of ICG-NIR in oesophageal cancer. For example, Hachey et al. (6) conducted a first-in-human study to assess the safety and feasibility of an intraoperative ICG-NIR image-guided approach to lymphatic mapping in patients with oesophageal cancer. They found that NIR lymphatic mapping was safe and could accurately identify regional lymph nodes. Similarly, Wang et al. (16) found that ICG-NIR imaging could successfully perform regional lymph node mapping in thoracic oesophageal cancer. However, although the abovementioned studies had positive results, the feasibility of using ICG-NIR imaging for guiding radical lymphadenectomy in oesophageal cancer is still under evaluation.

In this study, we prospectively enrolled patients with primary oesophageal cancer who underwent lymphadenectomy under ICG-NIR-guided imaging. We aimed to detect the effectiveness of ICG-NIR imaging during lymphadenectomy for oesophageal cancer.

## Materials and methods

### Patients' basic information

This prospective cohort study was approved by the ethics committee of The Affiliated Tumor Hospital of Nantong University in accordance with the Declaration of Helsinki. All the participants provided written informed consent before participating in the study.

The inclusion criteria were as follows: (1) patients who were diagnosed with primary oesophageal squamous cell carcinoma and were scheduled to undergo a minimally invasive Ivor Lewis esophagectomy, (2) patients aged 18–80 years, (3) patients with a tumour stage of cT_1_ to cT_3_, N−/+ and M0 at their preoperative evaluation and (4) patients with an ECOG score of 0 or 1.

The major exclusion criteria included being pregnant or breastfeeding, having a serious mental disorder, having a history of allergy to iodine or seafood and having a history of hyperthyroidism.

### Administration of ICG

**Figure 1:**
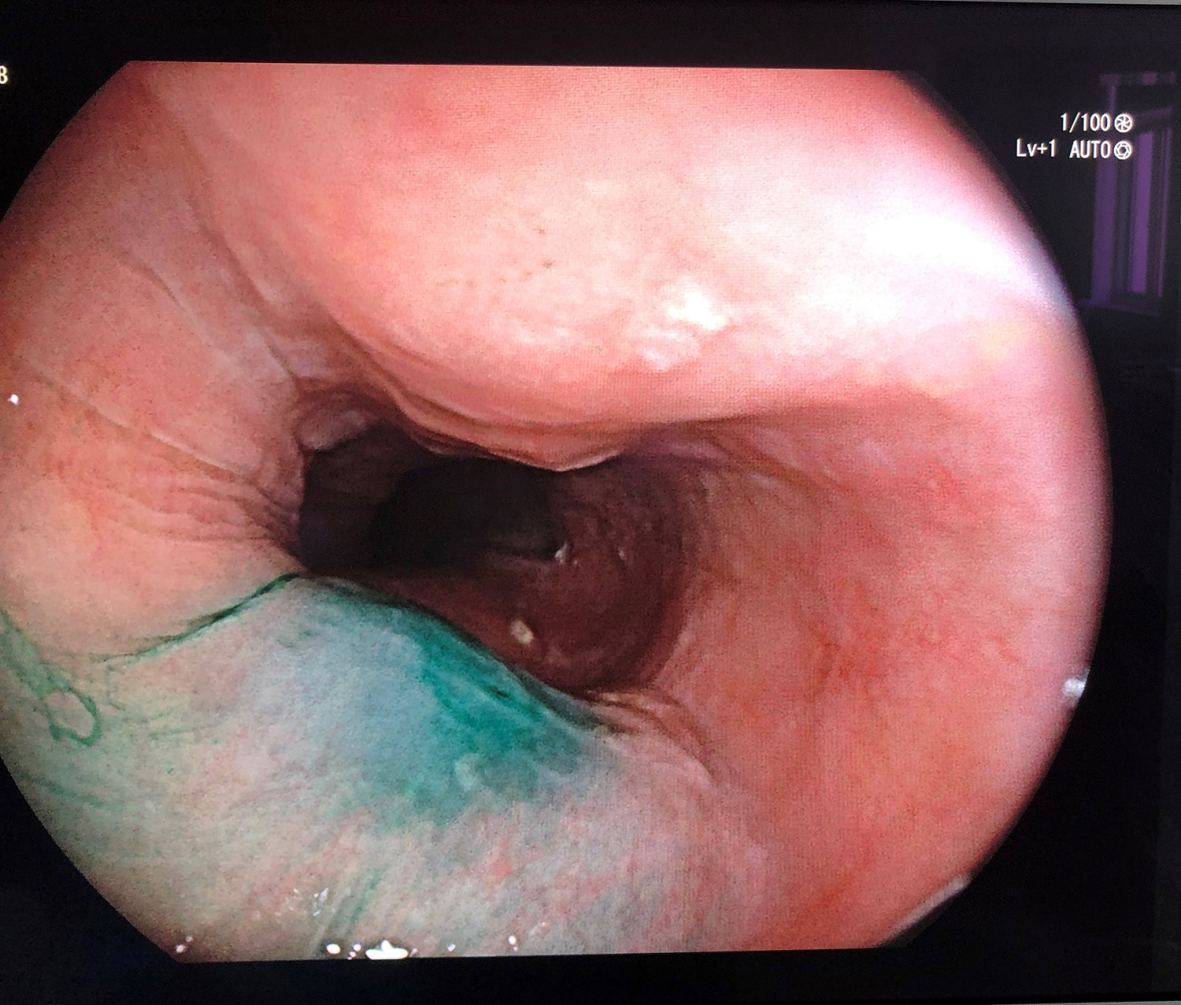
Endoscopic peritumoral ICG injection.

We purchased ICG powder from Dandong Yichuang Pharmaceutical Co. (Dandong, China) and diluted 25 mg of it in sterile water to create a 2.5-mg/ml solution. All the enrolled patients underwent endoscopic injection of ICG between 40 min and 23 h before surgery. A 0.5-ml volume of the prepared ICG solution was injected submucosally in the four quadrants around the tumour (Figure 1), providing a total volume of 2 ml. The ICG dose was determined based on previous studies (6, 17). The effectiveness of ICG-NIR fluorescence lymph node mapping was measured by comparing the positive rate of lymph nodes with fluorescence mapping to the overall positive rate of lymph nodes.

### Surgery and pathological examination

Preoperative examinations usually include blood routine, serum immunoglobulin, cellular immune test, liver function and renal function etc. All the enrolled patients underwent a minimally invasive Ivor Lewis esophagectomy and 2-field lymphadenectomy under the same surgical team. The extent of lymph node dissection was the extent in the conventional white light mode, and the number of lymph nodes was counted under the white light mode. Fluorescence-mapped lymph nodes were counted visually during fluorescence imaging. Nodal dissection was performed under fluorescence imaging visualisation, with the NIR signal shown in purple. Fluorescence activity was examined using an infrared camera. ICG^+^ or ICG^−^ nodes were recorded station by station. Real-time NIR fluorescence images were obtained using a NOVADAQ fluorescence surgical system (storz) with a 10-mm 30° NIR thoracoscopic camera. Adverse events, such as anaphylaxis, were monitored after the administration of ICG. Lymphatic stations were sent separately to the pathology department of our hospital.

All surgical lymph node specimens were fixed in formalin, stained with haematoxylin/eosin and examined by pathologists. The ICG^+^ and ICG^−^ nodes were microscopically evaluated and categorised as either metastatic or non-metastatic.

### Statistical analysis

The data in this study were analysed using SPSS 22.0 software. Categorical data were described in numbers (percentages) and compared using the Chi-squared test or Fisher's exact test. Quantitative data were described as mean ± standard deviation and compared using a one-way analysis of variance. The diagnostic accuracy of ICG-NIR imaging for metastatic lymph nodes was determined using the standard epidemiological method of calculating the sensitivity, specificity, positive predictive value (PPV) and negative predictive value (NPV). A value of *P* < 0.05 was considered statistically significant.

## Results

### Patients' characteristics

Eighty-seven patients with primary oesophageal cancer were enrolled in this prospective study. Among them, 64 were male, and 23 were female. The mean age was 68.45 ± 7.03 years, and the mean body mass index was 23.78 ± 2.70 kg/m^2^. The time from ICG injection to surgery ranged from 40 min to 23 h. Most of the patients (66.67%, 58/87) underwent endoscopic ICG injection 2–6 h before surgery. The characteristics of the enrolled patients are shown in Table 1. No patients experienced ICG-related adverse events (e.g., allergic reactions, rashes or bleeding associated with ICG injection), and no cases of chylothorax were observed.

**Table 1:** The clinical and pathological characteristics of the study patients.

Characteristics	*N*	%
**Gender**		
Male	64	73.56
Female	23	26.44
Age, years		
<60	10	11.49
≥60	77	88.51
BMI, kg/m^2^		
<24	48	55.17
≥24	39	44.83
Clinical T stage		
T_1_–T_2_	45	51.72
T_3_	32	36.78
Clinical N stage		
N_0_	57	65.52
N+	30	34.48
Time of ICG injection to surgery, h		
≤2	19	21.84
2–6	58	66.67
≥6	10	11.49
Neoadjuvant therapy		
Yes	24	27.59
No	63	72.41

Note: BMI, body mass index; ICG, indocyanine green.

### Complications

Endoscopic peritumoral ICG injection was performed successfully in all patients. The 30-day mortality rate was 0%, and 20 (22.99%) patients experienced postoperative complications. Major complications included wound infection (5/87, 5.75%), pleural effusion (9/87, 10.34%), dysphonia (4/87, 4.60%), pneumonia (3/87, 3.45%) and anastomotic fistula (1/87, 1.15%).

### ICG NIR fluorescence lymph node mapping

**Figure 2:**
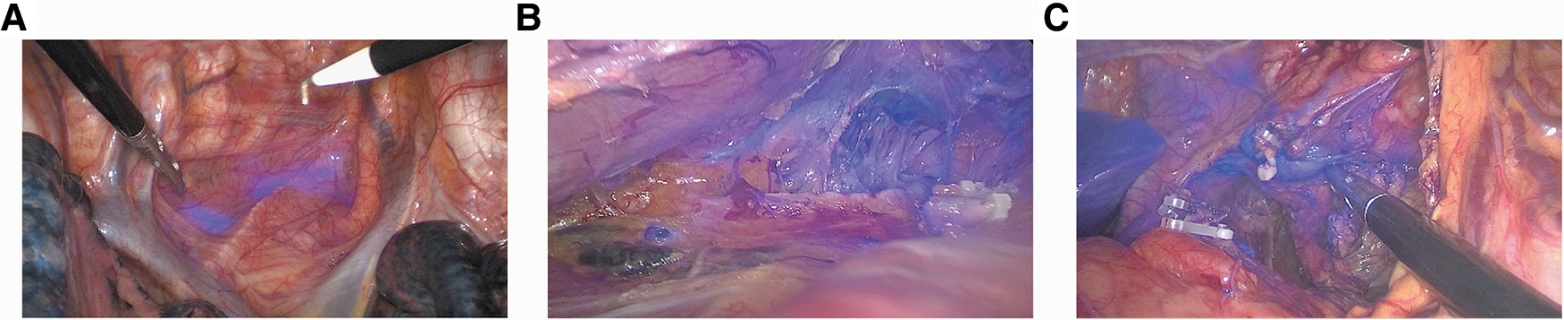
ICG-NIR image-guided identification of esophageal lymph nodes. (**A**) ICG^+^ periesophageal lymph nodes; (**B**) ICG^+^ paralaryngeal recurrent nerve lymph nodes and ICG^+^ lymphatic tract; (**C**) ICG^+^ left gastric lymph nodes and ICG^+^ lymphatic tract.

**Figure 3:**
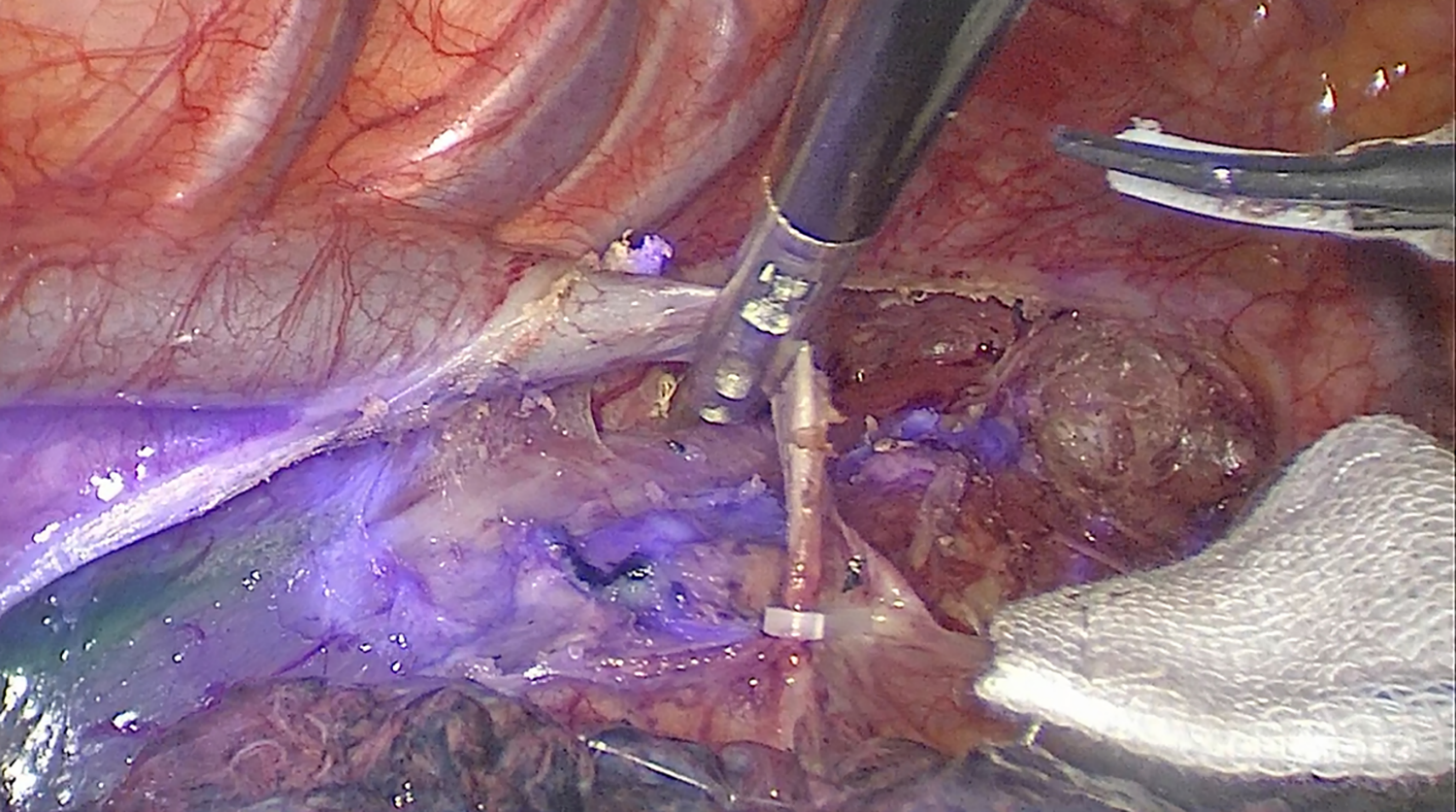
Significant NIR background directly on the esophageal specimen, particularly around the injection site.

We successfully detected lymphatic drainage spreading from the ICG injection site in all 87 patients (Figure 2). Significant NIR background directly on the oesophageal specimen was observed, particularly around the injection site (Figure 3). Dye extravasation into the posterior mediastinum was observed in 4 of the 87 patients, making it difficult to assess the lymph nodes directly. However, the marked lymph nodes could be identified. Thus, these 4 patients were also included in the statistical analysis. Interestingly, all 4 patients underwent endoscopic ICG injection ≥6 h before surgery.

A total of 576 lymph node stations were examined in the 87 patients. Among them, 557 (96.70%) stations showed at least one instance of positive ICG fluorescence. Of these 557 lymph node stations, 59 (10.59%) contained at least one metastatic node (Table 2). A total of 576 lymph node stations were detected with white light, of which 557 were fluorescent, and a total of 2,584 lymph nodes were removed. In all, 2,499 lymph nodes showed at least one positive instance of ICG fluorescence, and 85 lymph nodes showed negative ICG fluorescence. The mean number of lymph nodes for each patient was 29.70 ± 9.24 (median: 28, interquartile range: 23–35). Of the 2,584 lymph nodes, 2,528 (97.83%) were ICG^+^, among which, 84 (3.32%) were metastatic (Table 2). No metastatic nodes were ICG^−^ or belonged to an ICG^−^ lymph node station. The sensitivity of ICG-NIR imaging in the detection of the metastatic lymph nodes was 100%, and the specificity was only 2.24%. The overall PPV was only 3.32%, while the NPV was 100%.

**Table 2:** Lymphadenectomy specimens according to fluorescence and metastatic status.

	M^+^	M^−^	Total
**Lymph node stations**			
ICG^+^	59	498	557
ICG^−^	0	19	19
Total	59	517	576
**Lymph nodes**			
ICG^+^	84	2,444	2,528
ICG^−^	0	56	56
Total	84	2,500	2,584

Note: ICG, indocyanine green; M, metastatic node.

### ICG injection time

The time from ICG injection to surgery did not affect the number of harvested lymph nodes. Patients who underwent ICG injection ≥6 h before surgery appeared to have higher numbers of harvested lymph nodes (32.90 ± 14.10) than those who received an injection of ICG ≤2 h before surgery (29.70 ± 7.95) or 2–6 h before surgery (29.14 ± 8.70). The difference between the three populations was statistically significant (*P* = 0.500).

## Discussion

In this study, we analysed the feasibility and safety of using ICG-NIR imaging to guide surgeons in lymph node dissection in oesophageal cancer. As is well known, a complete lymphadenectomy is important to ensure correct staging in the surgical treatment of oesophageal cancer. Our study found ICG-NIR-guided lymphatic mapping during oesophageal cancer surgery to be a feasible, safe and helpful technique. Endoscopic peritumoral ICG injection was successfully performed in all the 87 patients enrolled, with 96.70% of lymph node stations and 97.83% of lymph nodes exhibiting fluorescence. All metastatic nodes were found to be ICG^+^ nodes.

Previously, ICG-NIR imaging was often used to detect sentinel lymph nodes in solid tumours, demonstrating that ICG was more effective than other tracers (18). For example, Kawakami et al. (19) found that the identification rate of ICG-NIR imaging for sentinel lymph nodes in lung cancer surgery was as high as 72.7%, and the high accuracy rate of this technique could be maintained throughout the follow-up period. Fujita et al. (20) examined the usefulness of ICG-NIR imaging in sentinel lymph node detection in gastric cancer surgery and found sentinel lymph node mapping using ICG fluorescence imaging to be highly accurate.

Numerous studies have attempted to use ICG-NIR imaging to guide radical lymphadenectomy for gastrointestinal tumours. Chen et al. (13) conducted a randomised study to investigate the safety and efficacy of ICG-NIR imaging during laparoscopic D_2_ lymphadenectomy in patients with gastric cancer. They found that ICG-NIR imaging significantly increased the number of lymph node dissections and reduced lymph node non-compliance (13). Kwon et al. (21) evaluated the role of ICG-NIR imaging as an intraoperative tool for achieving complete lymph node dissection in robotic radical gastrectomy in gastric cancer. In their study, the mean number of overall lymph nodes retrieved was higher in an NIR group than in a control group (48.9 vs. 35.2, *P *< 0.001), with a significantly higher number of lymph nodes retrieved at stations 2, 6, 7, 8 and 9 (21). Lan YT et al. (22) confirmed the feasibility of ICG-NIR imaging in robotic gastrectomy for gastric cancer. They found that all metastatic lymph nodes were located in the lymph node stations detected by ICG-NIR imaging. Similarly, a prospective trial by Baiocchi et al. (23) also discovered that ICG-NIR imaging-guided lymphadenectomy possessed a high feasibility rate, with considerable ease of use in gastric cancer. The safety and feasibility of ICG-NIR imaging-guided lymphadenectomy were also confirmed in patients with oesophageal cancer, as described in a previous study by Hachey et al. (6). Puccetti, F et al. (24) reported that the use of neoadjuvant radiotherapy had no significant effect on the percentage of ICG^+^ lymph node in the treatment of gastric cancer. And Zhang, L et al. (25) also reported that neoadjuvant radiotherapy had no effect on the percentage of ICG^+^ lymph node in breast cancer treatment.

In the present study, we also found that ICG-NIR imaging-guided lymphadenectomy was a safe method, and no ICG-related adverse event was observed. Major postoperative complications included wound infection, pleural effusion, dysphonia, pneumonia and anastomotic fistula, which was similar to previous studies (16, 26). Compared with previous research, the injection of ICG did not increase the rate of postoperative complications (26). Iwamoto, M. et al. (27) found that ICG could reduce the risk of complications such as anastomotic leakage in colorectal resection and Tokumaru, S. also reported that no complications were found using ICG and this method can be a powerful tool to avoid thoracic duct injury during esophageal cancer surgery (28).

Endoscopic peritumoral ICG injection was successfully performed in all 87 enrolled patients in this study, with 96.70% of lymph node stations and 97.83% of lymph nodes exhibiting fluorescence. In previous studies, the median number of lymph nodes retrieved in traditional 2-field lymphadenectomy for oesophageal cancer was 24 (interquartile range: 18–30) (29), while in the present study, ICG-NIR imaging-guided 2-field lymphadenectomy seemed to retrieve a higher number of lymph nodes (median: 28, interquartile range: 23–35). Thus, ICG-NIR imaging may increase the number of lymph node dissections during laparoscopic lymphadenectomy in patients with oesophageal cancer, as demonstrated by Chen et al. (13). However, this result should be confirmed by a larger two-arm randomised study. The key finding of our study is that all the retrieved metastatic nodes were ICG^+^ nodes; no metastatic nodes were ICG^−^ or belonged to an ICG^−^ lymph node station. Similar results were obtained by Baiocchi et al. (23), 22, Lan YT et al. (22) and Kwon et al. (21) in their respective studies.

In Wang's (16) research, the PPV of ICG-NIR imaging in metastatic lymph node detection was 5.89%, and the NPV was 94.38% in oesophageal cancer. In Park's study, the NPV of ICG-NIR imaging in the detection of nodal metastasis was 100% for the right recurrent laryngeal nerve chain and 98.2% for the left recurrent laryngeal nerve chain in T_1_ oesophageal cancer (30). Consistent with these studies, we also demonstrated that ICG-NIR imaging in metastatic lymph node detection had very high sensitivity and NPV, indicating that ICG^−^ lymph nodes were not the dominant region of tumour lymphatic drainage. The low PPV and specificity observed in this study indicated that ICG-NIR imaging was unable to identify metastatic lymph nodes specifically. Thus, ICG-NIR imaging could only reflect lymphatic drainage and could not improve the detection of metastatic lymph nodes.

The most suitable ICG injection time before surgery for regional lymph node identification in gastrointestinal tumours has not been determined. In Chen's (13) study, patients underwent endoscopic ICG injection 1 day before surgery, while in Wang's (16) research, ICG injection was administered 30 min before surgery (16). In a study by Romanzi et al. (31), patients underwent esophagogastroduodenoscopy for peritumoral submucosal ICG injection 18 h before surgery. In the present study, the time from ICG injection to surgery ranged from 40 min to 23 h. According to the time from ICG injection to surgery, we divided the enrolled patients into three groups (i.e., ≤2, 2–6 and ≥6 h) and found that the time from injection to surgery did not affect the number of harvested lymph nodes. However, most of the patients (66.67%) in this study underwent endoscopic ICG injection 2–6 h before surgery, which might have caused selection bias. Thus, further prospective studies are needed to confirm the optimal time for ICG injection.

In future studies, we believe that research on the effectiveness of ICG-NIR fluorescence lymph node localisation should be conducted extensively in a variety of tumours. To establish an optimised protocol for ICG injection, different doses, concentrations and times of ICG injection should be tested to determine the optimal times and doses in different tumours. At the same time, the ICG-NIR fluorescence lymph node localisation technique will be further improved to achieve more effective detection of metastatic lymph nodes.

The present study has several limitations. First, it was a single-centre study with a small sample size. A multi-centre with a larger sample size is needed to confirm the conclusions of this research. Second, most of the patients in this study received an endoscopic injection of ICG 2–6 h before surgery. This might have caused a selection bias in determining the optimal ICG injection time.

## Conclusions

This study revealed that ICG-NIR imaging during oesophageal cancer surgery can enhance lymph node visualisation during surgery. It is a feasible, safe and helpful technique for lymphadenectomy.

## Data Availability

The original contributions presented in the study are included in the article/Supplementary Material, further inquiries can be directed to the corresponding author.
